# Allogeneic mesenchymal stem cell as induction therapy to prevent both delayed graft function and acute rejection in deceased donor renal transplantation: study protocol for a randomized controlled trial

**DOI:** 10.1186/s13063-017-2291-y

**Published:** 2017-11-16

**Authors:** Qipeng Sun, Liangqing Hong, Zhengyu Huang, Ning Na, Xuefeng Hua, Yanwen Peng, Ming Zhao, Ronghua Cao, Qiquan Sun

**Affiliations:** 1 Department of Renal Transplantation, Lingnan Hospital, The Third Affiliated Hospital, Sun Yat-sen University, Kaichuang Road 2693, Huangpu District, Guangzhou, 510530 People’s Republic of China; 2 Cell-gene Therapy Translational Medicine Research Center, The Third Affiliated Hospital, Sun Yat-sen University, Tianhe Road 600, Guangzhou, 510630 People’s Republic of China; 3 Department of Renal Transplantation, Zhujiang Hospital, Southern Medical University, Gongye Road 253, Guangzhou, 510280 People’s Republic of China; 4 Department of Renal Transplantation, The Second Affiliated Hospital, Guangzhou Traditional Chinese Medicine University, Inner Ring Road 55, University City, Guangzhou, 510280 People’s Republic of China

**Keywords:** Mesenchymal stem cell, Renal transplantation, Delayed graft function, Acute rejection

## Abstract

**Background:**

Using kidneys from deceased donors is an available strategy to meet the growing need of grafts. However, higher incidences of delayed graft function (DGF) and acute rejection exert adverse effects on graft outcomes. Since ischemia-reperfusion injury (IRI) and ongoing process of immune response to grafts are the major causes of DGF and acute rejection, the optimal induction intervention should possess capacities of both repairing renal structure injury and suppressing immune response simultaneously. Mesenchymal stem cells (MSCs) with potent anti-inflammatory, regenerative and immune-modulatory properties are considered as a candidate to prevent both DGF and acute rejection in renal transplantation. Previous studies just focused on the safety of autologous MSCs on living-related donor renal transplants, and lack of concomitant controls and the sufficient sample size and source of MSCs. Here, we propose a prospective multicenter controlled study to assess the clinical value of allogeneic MSCs in preventing both DGF and acute rejection simultaneously as induction therapy in deceased-donor renal transplantation.

**Methods/design:**

Renal allograft recipients (n = 100) will be recruited and divided into trial and control groups, and 50 patients in the trial group will be administered with a dose of 2 × 10^6^ per kilogram human umbilical-cord-derived MSCs (UC-MSCs) via peripheral vein injection preoperatively, and a dose of 5 × 10^6^ cells via renal arterial injection during surgery, with standard induction therapy. Incidences of postoperative DGF and biopsy-proved acute rejection (BPAR) will be recorded and analyzed. Additionally, other clinical parameters such as baseline demographics, graft and recipient survival and other severe postoperative complications, including complicated urinary tract infection, severe pneumonia, and severe bleeding, will be also assessed.

**Discussion:**

This study will clarify the clinical value of UC-MSCs in preventing DGF and acute rejection simultaneously in deceased-donor renal transplantation, and provide evidence as to whether allogeneic MSCs can be used as clinically feasible and safe induction therapy.

**Trial registration:**

ClinicalTrials.gov, NCT02490020. Registered on 29 June 2015.

**Electronic supplementary material:**

The online version of this article (doi:10.1186/s13063-017-2291-y) contains supplementary material, which is available to authorized users.

## Background

Renal transplantation is the definitive solution for patients with end-stage renal failure [1]. Growing need for renal transplantation far exceeds the supply of donor organs and the shortage is becoming more severe for the increasing numbers of patients listed for transplantation. According to the Eurotransplant report, only 1788 transplant kidneys were available for 10,689 patients on the waiting list in 2014 [2]. To overcome this problem, the use of deceased donors is becoming the major source of transplants. However, in the process of deceased-donor renal transplantation, prolonged ischemic time from donor harvest to kidney reperfusion in recipients inevitably results in higher incidences of delayed graft function (DGF) and acute rejection, and causes adverse effects on graft outcome [3–6]. DGF is identified as a strong risk factor for subsequent chronic allograft dysfunction and shortened allograft survival, but current prevention methods such as the application of erythropoietin (EPO) and hepatocyte growth factor (HGF) do not demonstrate significant clinical benefit in decreasing DGF [7–11]. Moreover, prolonged ischemic time leads to a significantly earlier and greater onset of acute rejection, which also exerts an adverse effect on graft survival. Therefore, interventions aimed at both decreasing DGF and suppressing acute rejection may be a promising way to improve early graft function.

DGF is primarily a consequence of pre-transplant injury and immune responses after reperfusion. Severe ischemia-reperfusion injury (IRI) and acute kidney injury (AKI) adversely affect renal structure such as early tubular atrophy and interstitial fibrosis during transplantation using deceased donors, which is caused by accumulation of metabolic waste products such as nitric oxide (NO), superoxide anion and hydrogen peroxide to initiate oxygen-radical-induced injury and apoptosis of cell membrane [12]. As for acute rejection, mediators of the innate and adaptive immunity such as toll-like receptors (TLRs) are activated to trigger T cell clonal expansion and differentiation of effector cells, which modulate the complement cascade to aggravate donor kidney injury [13]. Hence, the optimal therapy to prevent both DGF and acute rejection for renal transplantation using deceased donors should possess capacities of both repairing renal structure injury and suppressing immune response simultaneously. However, no current induction therapies achieve this goal.

Mesenchymal stem cells (MSCs), represent significant anti-inflammatory, tissue repair and immune-modulatory properties, and suggest a novel cell-based approach in the renal transplantation. Effects of MSCs have been explored in many preclinical models of AKI [14]. MSCs can secrete numerous growth factors such as insulin-like growth factor 1 (IGF-1) and vascular endothelial growth factor (VEGF), disrupt inflammatory response in the injured kidney accounting for improving recovery from IRI [15, 16]. In the experimental models, MSCs have also been shown to inhibit T cell proliferation, modulate B cell functions and suppress natural killer cytotoxic effects, which may be applied to inhibit allograft rejection and induce transplant tolerance [17]. Therefore, MSCs have potential benefits to prevent both DGF and acute rejection. However, few clinical data on MSCs applied to transplantation have been published, in which potential disadvantages of results had negative effects on clinical application of MSCs, due to lack of concomitant controls and sufficient sample size and source of MSCs [18–20].

In our previous study, we performed human umbilical-cord-derived MSCs (UC-MSCs) to kidneys from donors after cardiac death (DCD) and found a significant effect on reduction of both antibody-mediated rejection and AKI [21]. Now, we will carry out a prospective multicenter controlled study to clarify the clinical value of UC-MSCs as induction therapy to prevent both DGF and acute rejection in deceased-donor grafts. Here, we propose the protocols of this study.

## Methods/design

### Objectives

The objective of this study is to assess the clinical value of human UC-MSCs as induction therapy to prevent both DGF and acute rejection via different ways of administration in deceased-donor renal transplantation.

### Endpoints

#### Primary endpoints

The primary endpoints of this study include DGF during one week post transplantation, and biopsy proven acute rejection (BPAR) over one year.

#### Secondary endpoints

The secondary endpoints are severe opportunistic infections related to opportunistic infection and pulmonary and urinary tract infection over one year postoperatively.

### Study design

This is a prospective multicenter controlled study including three kidney transplant institutions (the third affiliated hospital of Sun Yat-sen University, Zhujiang hospital of Southern Medical University, and the second affiliated hospital of Guangzhou Traditional Chinese Medicine University). The protocol has been approved by the Ethics Committee of Human Study at the three institutions, which were established according to the Operational Guidelines for Ethics Committees that Review Biomedical Research developed by the World Health Organization (WHO) [22].

No organs from executed prisoners will be used in the study, and procurement of kidneys from all donors will be conducted in accordance with the WHO principles, Declaration of Helsinki and Istanbul declaration [23, 24]. Organ donation and recovery will be conducted by organ procurement organizations in the three kidney transplant institutions, which were established by the National Health and Family Planning Commission of China. Before procurement, written consent will be obtained from the donor’s immediate family, agreeing to donation of the kidney and withdrawal of life support. The obtained consent for donation will be then reported to the Organ Donation Committee, which supervises the donation process.

**Fig. 1:**
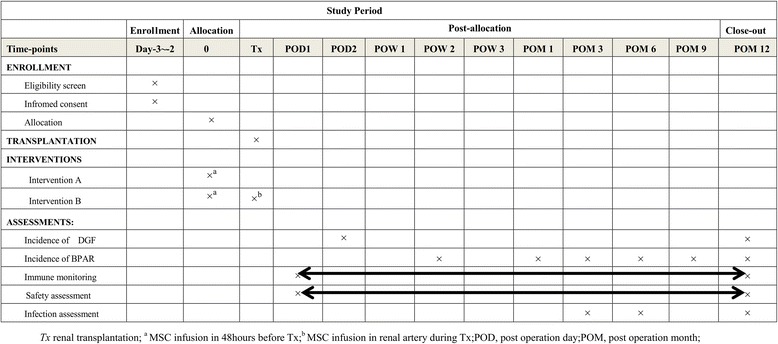
Summary of enrollment, interventions, assessments, and timing for measurements (Standard Protocol Items: Recommendations for Interventional Trials (SPIRIT)). DGF, delayed graft function; BPAR, biopsy-proven acute rejection

All the participants in this study will provide informed, written consent to undergo this study. Participants receiving renal transplantation with deceased-donor grafts will be recruited and randomly divided into two groups. The trial group will undergo peripheral vein injection of human UC-MSCs in the 48 hours before the operation, and renal arterial injection during the operation. The control group will not receive UC-MSC treatment. All the participants will have standard induction therapy. All the participants will be recruited from the three transplant units and meet the eligible criteria. The Standard Protocol Items: Recommendations for Interventional Trials (SPIRIT) Figure is provided in Fig. 1.

### Eligibility criteria

#### Inclusion criteria

1. Female or male aged between 18 and 60 years
2. Subjects who conform to the indications for renal transplantation
3. Subjects who are willing to participate in the study and have provided written informed consent prior to any study procedure
4. Subjects receiving a first kidney graft from deceased donors

#### Exclusion criteria

1. Subjects who are lost to follow-up.
2. Contraindications to undergoing renal transplantation, such as patients with evidence of active infection, suffering from hepatic failure, an active autoimmune disease, documented HIV infection, active hepatitis B, hepatitis C or tuberculosis (TB), cytomegalovirus (CMV), BK virus, aspergillosis, histoplasmosis or mycobacteria other than TB, or a previous bone marrow transplant.
3. Occurrence of severe adverse events, such as toxic effects, CMV reactivation, and post-transplant lymphoproliferative disease.
4. Double organ-transplant recipients.
5. Treatment with any investigational drug used after transplantation.
6. Malignancy within the past 5 years according to current transplantation inclusion criteria.
7. Known recent drug abuse.
8. Recipients of ABO blood-type incompatible transplants.

### Treatment methods

#### UC-MSC treatment

A target of 2 × 10^6^ UC-MSCs per kilogram body weight will be injected via the peripheral vein during the 48 hours before renal transplantation. The dose of UC-MSCs administered via the renal artery during the implantation surgical procedure is 5 × 10^6^ cells.

#### Standard induction therapy

The standard induction therapy is described as follows: all recipients receive antithymocyte globulin (ATG) 50 mg for 3 days and methylprednisolone (MP) with a total amount of 2.0 g. Then, prednisone (Pred) is taken orally at the initial amount of 30 mg, and tapered at a dose of 2.5 mg per week, to the maintaining dose of 5 mg per day. Mycophenolate (MMF) is taken from that day after the operation at a dose of 1.0 g twice a day. Calcineurin inhibitors (CNI), (such as cyclosporine (CsA) or FK506) are given 3 days after the operation.

### Isolation of UC-MSCs

The human UC-MSCs used in this study will be isolated after birth with the written consent of the parents. The human UC-MSCs will be harvested in a total volume of 100–120 ml. The processing and expansion of the cells will take place at the Good Manufacturing Practice (GMP) Stem Cell Laboratory Facility of Sun Yat-sen University, as previously described [25]. The characterization of the final product will be determined by the flow cytometric analysis, which expresses CD90, CD73, CD105, CD44, and CD166. Before infusion, the human UC-MSCs will be subjected to aerobic, anaerobic and fungal cultures and tested for mycoplasma infection, and then confirmed to be sterile.

### Randomization and blinding

**Fig. 2:**
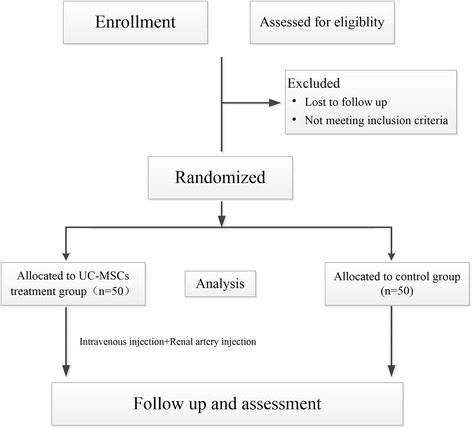
Overview of this trial procedure UC-MSCs, umbilical-cord-derived mesenchymal stem cells

The participants will be assigned to either the UC-MSCs treatment group or the control group in a 1:1 ratio using a block randomization method. A randomization list has been pre-generated. The participants will be blinded to the treatment group throughout the study (Fig. 2).

### Data collection and postoperative monitoring

Baseline clinical demographics of donors (such as age, gender, body mass index, medical history, infection status, serum creatinine, causes of death, time of ICU, urine volume per day, creatinine level at organ procurement, time of warm and cold ischemia, use of vasoactive drugs and cardiopulmonary resuscitation) will be documented. For recipients, clinical data include age, gender, medical history, current medication, blood type, previous blood transfusions, PRA, infection status, physical examination, laboratory examinations, methods of dialysis, and duration of dialysis. HLA mismatching numbers of donor-recipient, complement-dependent cytotoxicity (CDC), time-zero donor kidney biopsy will also be collected. All immunosuppressive and other drugs used and dosages administered will be recorded during the study. Follow up will be in accordance with the assessment schedule in Table 1.

**Table 1:** Assessment schedule for recipients

Procedure	Time point											
	BL	Tx	POD1	POD2	POW1	POW2	POW3	POW4	POW12	POW24	POW36	POW48
Medical history	×											
Transplantation information		×										
Concomitant medication	×	×			×	×	×	×	×	×	×	×
Physical examination	×		×	×	×	×	×	×	×	×	×	×
Blood sample test	×		×	×	×	×	×	×	×	×	×	×
Urinalysis	×		×	×	×	×	×	×	×	×	×	×
Viral load CMV and BK virus									×	×		×
Urine volume			×	×	×	×	×	×	×	×	×	×
Renal color ultrasound			×		×				×	×		×
Time-zero and protocol biopsies		×				×						×
CNI concentration					×	×	×	×	×	×	×	×
Safety assessment	×	×	×		×	×	×	×	×	×		×
Infection assessment									×	×		×
Chest CT scan	×								×	×		×

*BL* baseline, *Tx* renal transplantation, *POD* post operation day, *POW* post operation week, *CMV* cytomegalovirus, *CNI* calcineurin inhibitor, *CT* computed tomography

DGF is defined as urine output < 1000 ml/day in first 48 hours or failure of serum creatinine to decrease by 10% in the first 48 hours postoperatively, according to Shoskes [26]. BPAR is classified according to the Banff 2013 classification [27]. Renal color ultrasound will be performed at 1 day, 7 days, 1 month, 3 months, 6 months, and 1 year postoperatively. Time-zero biopsy and protocol biopsies at 14 days and 12 months postoperatively will be performed. When acute rejection is suspected clinically, renal allograft biopsy will also be carried out. The incidence of BPAR at 2 weeks, 1 month, 3 months, 6 months, and 1 year after the operation will be recorded. For opportunistic infection, the incidence of CMV, EBV, and BK-viremia will be measured in blood samples, and pulmonary and urinary tract infection will be monitored at 3 months, 6 months, and 1 year after the operation.

Additionally, laboratory examinations such as routine blood and urine tests, tests of liver and renal function, and concentration of CNI will be assessed once a week in the 3 months after the operation, once every 2 weeks in months 3 to 6, and once a month in months 6 to 12.

Adverse side effects in the UC-MSC treatment group may include fever, uncommon arrhythmia and allergic reaction. Adverse events will be recorded and treated. The trial blood samples will be collected to examine safety.

We will make every effort to minimize missing data. Trial enrollment and follow-up procedures will be reviewed during bi-monthly conference calls. We will report rates of missing data for each outcome by study arm and send missing data reports to the sites.

### Sample size

The sample size was calculated based on our previous data showing that there was no BPAR in the MSC treatment group at 6 months after transplantation, compared with 16.7% of acute rejection in the control group [21]. Based on this preliminary study, we calculated that 44 patients per arm will be required to achieve power of 90% with a two-sided significance level of *P* < 0.05. To account for possible dropouts (10%), the target number of patients was, therefore, set at 50 per arm (100 in total) (see Additional file 1).

### Statistical analysis

Parametric (e.g., analysis of variance (ANOVA)) and non-parametric (e.g., Kruskal-Wallis test) statistical tests will be applied using the SPSS version 21 (SPSS Inc., Chicago, IL, USA) for the data analysis. Multi-level regression analysis will be used to examine differences between trial arms for the endpoints. The logistic model will be applied for binary endpoints, and linear regression will be for continuous endpoints. All analyses will follow intention-to-treat principles and a pre-specified analysis plan. As for missing data values, we will apply mean imputation and regression imputation where rates are low, and consider multiple imputations where they exceed 10%. Since multiple tests are performed simultaneously, the Bonferroni correction will be made during analysis to determine the critical *P* value for significance. *P* values < 0.05 will be considered as statistically significant. The SPIRIT checklist is provided in Additional file 2.

## Discussion

Prolonged ischemia time during renal transplantation using deceased donors causes more severe events related to ischemia and reperfusion injury (IRI) and acute kidney injury (AKI), which inevitably increase the incidence of DGF and acute rejection [3]. An appeal has been made for the application of novel induction therapy, which should be an optimal strategy to minimize the risks of both DGF and acute rejection simultaneously.

Previous studies have revealed that local cytokines, Toll-like receptors (TLRs), adhesion molecules such as intercellular cell adhesion molecule-1 (ICAM-1), vascular cell adhesion molecule-1 (VCAM-1), and the complement system induced by ischemia and reperfusion injury (IRI) can lead to inflammatory response, and cause kidney tubular epithelial cell death and lysis [28, 29]. At the same time, graft immunogenicity associated with acute rejection after transplantation, is increased because of upregulation of T cell costimulatory molecules such as CD80 and CD86, which can serve as antigen-presenting cells (APCs) for T cell activation [28]. Therefore, the most adequate intervention to prevent both DGF and acute rejection simultaneously should possess capabilities of inhibiting inflammatory response and repairing tissue injury, and suppressing immune response.

MSCs, as multi-potent progenitor cells, can not only inhibit inflammatory and immune responses [30, 31], but also have the capabilities of anti-fibrotic and regenerative treatment [32]. MSCs have been reported to strongly inhibit T cell proliferation, induce T cell division arrest and inhibit natural killer (NK) cell proliferation by combination factors, including IL-10 and transforming growth factor (TGF) [33–35]. Additionally, MSCs have also been found to induce regulatory T cells (Tregs) and decrease immune response [36]. These specialized characteristics of MSCs have made them attractive for use in inflammatory and immunity disorders [32, 37, 38]. MSCs have also been confirmed to have the capability of ameliorating tissue damage in response to injury and disease. In animal models, MSCs decrease fibrosis in the kidney, owing to the downregulation of collagen type I and III, TGF, and Smad2 expression by MSCs, and the tissue regeneration of MSCs [39]. Additionally, MSCs have been shown to consist of vascular endothelial growth factor (VEGF), which can enhance proliferation of endothelial cells to reduce the microvascular injury in the ischemic kidney after the injection of MSCs [40].

However, few studies have assessed the safety and efficacy of MSCs in the clinical setting [18–20]. In a recent study, patients administered with autologous MSCs had a lower incidence of acute rejection, opportunistic infection, and better estimated glomerular filtration rate at 1 year [19]. Despite benefits shown in recent MSC trials in renal transplantation, no studies on the simultaneous prevention effects of both DGF and acute rejection by MSCs from deceased donors have been found. Additionally, most studies have used autologous MSCs, which possess several key disadvantages due to lack of donor selection and availability of “off-the-shelf” clinical use without the delay required for expansion, compared to allogeneic MSCs [20]. In our study, we will use human UC-MSCs as induction therapy to assess the efficacy of preventing both DGF and acute rejection simultaneously after renal transplantation. The ultimate goal of this approach is to achieve a state of low incidence of DGF and acute rejection, and long-term transplant survival. Meanwhile, the safety and efficacy of human UC-MSCs will be confirmed to expand the source of MSCs.

Based on the above described mechanism of MSCs on tissue repair, anti-inflammatory and immune mediation, our hypothesis is that human UC-MSCs can prevent DGF in the early post-transplant phase. Moreover, we expect human UC-MSCs to ameliorate IRI and suppress immune response to decrease acute rejection of the graft, thereby avoiding chronic graft dysfunction. Limitations of our study include: (1) this is the first study on application of human UC-MSCs to prevent both DGF and acute rejection of deceased donor kidneys rather than living-related grafts, and the safety and efficacy of human UC-MSCs needs to be monitored; (2) although we identified a significant effect of human UC-MSCs on reduction of both antibody-mediated rejection and AKI in our previous study [21], we may have been underpowered to detect any significant differences in outcomes in this prospective multicenter controlled study.

Taken together, a positive outcome from allogeneic MSCs in terms of preserving renal function to decrease DGF, reducing acute rejection, and prolonging long-term survival will implicate a major advancement for deceased-donor renal transplantation.

### Trial status

The study is still recruiting at the time of submission.

## Additional files

Additional file 1: Sample size calculation. (DOCX 28 kb)

Additional file 2: SPIRIT 2013 checklist: recommended items to address in a clinical trial protocol and related documents. (DOC 150 kb)

## Abbreviations

AKI: Acute kidney injury
APCs: Antigen-presenting cells
ATG: Antithymocyte globulin
BPAR: Biopsy-proven acute rejection
BUN: Blood urea nitrogen
CDC: Complement-dependent cytotoxicity
CMV: Cytomegalovirus
CNI: Calcineurin inhibitors
CsA: Cyclosporine
DGF: Delayed graft function
EBV: Epstein-Barr virus
EPO: Erythropoietin
GMP: Good manufacturing practice
HGF: Hepatocyte growth factor
HIV: Human immunodeficiency virus
HLA: Human leukocyte antigen
ICAM-1: Intercellular cell adhesion molecule-1
IGF-1: Insulin-like growth factor-1
IL: Interleukin
IRI: Ischemia-reperfusion injury
MMF: Mycophenolate
MP: Methylprednisolone
MSCs: Mesenchymal stem cells
NK cell: Natural killer cell
NO: Nitric oxide
PRA: Panel-reactive antibody
Pred: Prednisone
TB: Tuberculosis
TGF: Transforming growth factor
TLRs: Toll-like receptors
Tregs: Regulatory T cells
UC-MSCs: Umbilical-cord-derived MSCs
VCAM-1: Vascular cell adhesion molecule-1
VEGF: Vascular endothelial growth factor
