# Supplementary material for: Allogeneic mesenchymal stem cell as induction therapy to prevent both delayed graft function and acute rejection in deceased donor renal transplantation: study protocol for a randomized controlled trial
Source: Trials. 2017 Nov 16;18:545. doi: 10.1186/s13063-017-2291-y (PMC5689202; doi:10.1186/s13063-017-2291-y)
Supplement: Supplementary file 1 — Sample size calculation. (DOCX 28 kb) [file 13063_2017_2291_MOESM1_ESM.docx]

**Sample size**

The sample size was calculated based on our previous data showing that there was no BPAR in MSC treatment group at 6 months after transplantion,compared with 16.7% of acute rejection in the control group[Peng Y, Ke M, Xu L, Liu L, Chen X, Xia W, et al. Donor-derived mesenchymal stem cells combined with low-dose tacrolimus prevent acute rejection after renal transplantation: a clinical pilot study. Transplantation.2013;95(1):161-8]. Based on this preliminary study, we calculated that 44 patients per arm would be required to achieve a power of 90% with a two-sided significance level of p < 0.05. To account for possible dropouts (10%), the target number of patients was, therefore, set at 50 per arm (100 in total)

The calculated formula was as following:


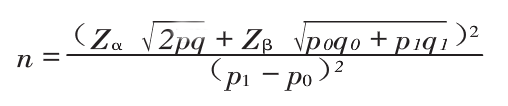


Here, ‘n’ is the number of patients in each arm;

‘*Z*_α_’ represents the value of α error；

‘*Z*_β_’ represents the value of βerror；

*p*_0_  represents the incidence of acute rejection in control group;

*p*_1_ represents the incidence of acute rejection in treatment group;

*q*_0 =_ 1-*p*_0_, q_1=_1-p_1_

*p*= (*p*_0_+*p*_1_)/2, q=1-*p*

*Z*_α_=.1.96, *Z*_β_=1.282

To account for possible dropouts (10%), the target number of patients was therefore set at 30 per arm (60 in total).
